# Dietary Betaine Improves Immune Function, Antioxidant Status, Immune‐Related Signaling Molecules, and Disease Resistance in Fish Species

**DOI:** 10.1155/anu/4596572

**Published:** 2025-12-14

**Authors:** Morteza Yousefi, Taravat Molayemraftar, Seyyed Morteza Hoseini, Hamed Ghafarifarsani

**Affiliations:** ^1^ Department of Veterinary Medicine, RUDN University, 6 Miklukho-Maklaya Street, Moscow, 117198, Russia, rudn.ru; ^2^ Department of Clinical Sciences, Faculty of Veterinary Medicine, Shahid Chamran University of Ahvaz, Ahvaz, Iran, scu.ac.ir; ^3^ Inland Waters Aquatics Resources Research Center, Iranian Fisheries Sciences Research Institute, Agricultural Research, Education and Extension Organization, Gorgan, 4915677555, Iran, ifsri.ir; ^4^ Department of Animal Science, Chaharmahal and Bakhtiari Agricultural and Natural Resources Research and Education Center, Agricultural Research, Education and Extension Organization (AREEO), Shahrekord, Iran, areo.ir

**Keywords:** aquaculture nutrition, betaine supplementation, biochemical pathways, fish physiology, immunity

## Abstract

Phytochemicals derived from plants have attracted attention as feed additives in aquaculture due to their natural bioactive properties. These compounds have a wide range of benefits such as improving fish growth, increasing feed efficiency, and enhancing immunity. As natural alternatives to synthetic chemicals and antibiotics, phytochemicals contribute to disease resistance, reducing oxidative stress, and promoting gut health in aquatic animals. Betaine, a natural compound with osmotic and metabolic functions, has become a suitable feed additive in aquaculture due to its potential benefits for fish health. The role of betaine in maintaining cellular osmotic balance and enhancing physiological processes such as protein synthesis has made it an effective dietary supplement for enhancing fish growth. Recent studies indicate that this compound not only supports growth performance but also plays a fundamental role in enhancing fish immunity. Betaine enhances the innate defense mechanisms of fish against pathogens by increasing the activity of immune cells, especially macrophages, regulating cytokine production, and reducing inflammation. In addition, betaine’s antioxidant properties reduce oxidative stress and improve immune signaling, helping fish maintain optimal immune function even in stressful environments. This comprehensive review aims to investigate the multifaceted role of betaine in improving fish immunity and antioxidant responses and the mechanisms associated with these roles.

## 1. Introduction

Aquaculture has experienced significant growth in recent decades to provide high‐quality animal protein. This level of growth is particularly observed in Asian countries (91.6% of global production in 2019) and regions where aquaculture has become a key component of the economy [1]. With the increasing global demand for aquatic products and decreasing fish stocks in the wild, fish farming has become essential to meet food needs. However, this expansion brings several challenges, including disease outbreaks, environmental stress, fluctuations in water quality, and reduced production efficiency. These factors can lead to stunted growth, higher mortality, and reduced productivity in fish farms [2–9]. Strengthening the immune system and reducing environmental stress are crucial to address these issues in fish [10, 11]. Pathogens such as bacteria, viruses, and parasites continuously threaten farmed fish, while environmental conditions such as salinity, temperature changes, and poor water quality cause further stress [12]. In the past decades, antibiotics and chemicals have been used to control diseases and enhance fish growth. However, concerns about antibiotic resistance, environmental damage, human health risks, and the potential transfer of these substances into the food chain have increased the search for safer and natural alternatives [13–15]. Hence, natural compounds derived from plants and other sources have emerged as promising alternatives [8, 15–19]. Betaine, an organic compound found in plants, especially in sugar beets, plays a key role in regulating cellular processes due to its biochemical and physiological properties [20–22]. As a natural osmolyte, betaine helps fish cope with environmental stress by maintaining osmotic balance and regulating water and electrolytes in cells, especially in salinity and temperature fluctuations [21, 23].

In addition, betaine acts as a methyl group donor in metabolic reactions, facilitating protein synthesis and other vital biological functions [24–27]. These properties have made betaine a popular dietary supplement for promoting fish growth and health. In addition to its role in improving growth and feed efficiency, betaine also strengthens the immune system of fish and increases their resistance to pathogens by regulating immune cell activity, reducing inflammation, and producing antimicrobial molecules [28–30]. Due to these immunogenic properties, betaine is increasingly considered a natural and safe additive for aquaculture that can be, to some extent, a potentially effective alternative to antibiotics and chemicals. This article aims to comprehensively review the immunogenic effects of betaine on fish and the molecular mechanisms involved. This insight can guide researchers and industry professionals in utilizing the unique properties of betaine to promote sustainable fish farming practices.

## 2. Structure and Biochemical Importance of Betaine

Betaine (C_5_H_11_NO_2_) is an organic compound classified as an amino acid that is naturally found in plants and animals. The chemical structure of betaine includes a quaternary amine group (N(CH_3_)_3_) and a carboxylate group (─COO─), which gives betaine its zwitterionic properties. This property means that betaine carries both positive and negative charges at physiological pH, allowing it to remain electrically neutral [23, 31]. Specifically, betaine is a methylated derivative of glycine, commonly known as N,N,N‐trimethylglycine [32]. Due to the methyl groups attached to it, betaine acts as a methyl donor in various biochemical processes and plays a key role in methylation reactions, especially in methionine synthesis [23, 33].

One of the key functions of betaine is that it is a natural osmolyte, which helps cells maintain osmotic balance. This ability to absorb and retain water allows betaine to protect cells from environmental stresses such as high salinity or temperature changes [21]. Its zwitterionic structure also ensures betaine’s stability in various environments, making it a widely used compound among various organisms as a cell protector against osmotic stress [34]. Betaine’s role as a methyl group donor is crucial in cellular biochemistry. Betaine facilitates the transfer of methyl groups to molecules such as homocysteine, converting it to methionine through the methylation cycle. This process is essential for maintaining metabolic balance, supporting protein synthesis, and regulating gene expression [35].

To play these vital roles, betaine must first be effectively absorbed from the gastrointestinal tract and into the bloodstream. Betaine is absorbed primarily in the early part of the small intestine, particularly in the duodenum and jejunum, by passive diffusion. This process is influenced by the concentration of betaine in the intestinal lumen and the physiological characteristics of the intestinal mucosa. Studies have shown that betaine enters intestinal epithelial cells freely and without the need for specific transporters, although, in some species, the role of osmolyte transporters, such as BGT‐1, has also been suggested. After absorption, betaine is transported via the bloodstream to the liver, where it plays a role in methylation reactions and the regulation of cellular osmolarity. The extent of betaine absorption can be influenced by nutritional factors such as the presence of other osmolytes (such as choline and glycine), the physiological state of the animal, and the form of the supplement consumed. In general, betaine has high bioavailability and is effectively absorbed in most animal species [27, 36, 37]. Therefore, due to its unique properties, betaine is an essential metabolite in living organisms.

## 3. The Importance of Betaine as a Feed Supplement in Aquaculture

Betaine is of considerable importance as a feed supplement in the aquaculture industry (Table 1). Its multifaceted properties help to increase growth, improve health, increase resistance to environmental stress and diseases, and optimize feed efficiency. By using betaine, aquaculture operations can be more stable and productive, and it is also a safe and secure alternative to chemicals and antibiotics. Therefore, these benefits can position betaine as a nutritional supplement to increase the performance of fish farms and improve the quality of final products.

**Table 1:** Effect of betaine dietary supplements on the performance of various fish species in aquaculture.

Fish species	Doses/duration	Parameters investigated	Optimal dose of betaine	References
Nile tilapia (*Oreochromis niloticus*)	500 and 1000 mg/kg of feed	CAT activity 🡩	500 mg/kg	[38]
Nile tilapia (*O. niloticus*)	0.50, 1.00, and 2.00 g/kg/ 100 days	CAT activity 🡩 growth performance 🡩	0.50 g/kg	[39]
Nile tilapia (*O. niloticus*)	10 g/kg dry feed/42 days	Nonspecific immune parameters 🡩 MDA 🡫 CAT, SOD, GSH, and GPx 🡩	10 g/kg	[40]
Caspian trout, (*Salmo trutta*)	1.4% and 2.8% betaine supplementation/77 days	Lysozyme activity and total serum immunoglobulin 🡩 CAT, SOD, and GST 🡩	1.4%	[29]
Blunt snout bream (*Megalobrama amblycephala*)	0.6%, 1.2%, 1.8%/56 days	SOD, CAT, and GSH 🡩 MDA 🡫	1.2%	[41]
Rainbow trout (*Oncorhynchus mykiss*)	25% soybean‐1% betaine diet and 50% soybean‐2% betaine diet/54 days	Weight gain, specific growth rate, and feed intake 🡩 Feed conversation ratio and survival = Total saturated fatty acid 🡩 Monounsaturated fatty acid 🡫	1%	[42]
Rainbow trout (*O. mykiss*)	0.9% and 1.63% betaine	Stress challenge parameters (Cortisol in plasma and fin, glucose and lactate plasma levels, and malondialdehyde (MDA) in muscle) =	Not effective	[43]
Nile tilapia (*O. niloticus*)	0.3% concentrations of betaine/56 days	Serum interleukins, TNF‐ alpha, and IL‐10 levels 🡫 MDA 🡩	0.3%	[44]
Nile tilapia (*O. niloticus*)	5, 10, 15, 20, and 25 g/kg/56 days	Survival and hepatic composition = Weight gain, specific growth rate, and feed intake 🡩	5 g/kg	[45]
Grass carp (*Ctenopharyngodon idella*)	1.6, 3.2, 4.8, and 6.4 g/kg/60 days	Lysozyme activities (LZ), complement 3 (C3), and C4 contents and IgM contents 🡩	1.6 and 3.2 g/kg	[28]
Common carp, (*Cyprinus carpio*)	0.25% and 0.50% betaine in 30% protein diet/60 days	Specific growth rate, survival, and protein efficiency ratio 🡩 Food conversion rate 🡫	0.25%	[46]
Common carp, (*C. carpio*)	0.5%, 1%, and 2% betaine	Total antioxidant capacity (T‐AOC), antioxidant enzymes [serum catalase (CAT), superoxide dismutase (SOD), glutathione peroxidase (GPx)], and immune components [lysozyme, total immunoglobulin (Ig), myeloperoxidase (MPO), alternative complement activity (ACH50), nitroblue tetrazolium (NBT) and total protein in blood and total Ig, lysozyme, alkaline phosphatase (ALP) and protease in mucus 🡩 MDA 🡫	1% and 2 %	[47]
Common carp, (*C. carpio*)	0.25% and 0.50% betaine/60 days	Specific growth rate, survival, food conversion rate, and protein efficiency ratio 🡩	0.25%	[48]
Olive flounder (*Paralichthys olivaceus*)	0.1%, 0.5%, and 1.0% betaine	Lysozyme activity and serum bactericidal activity 🡩	0.5%, and 1.0%	[30]
Mandarin Fish (*Siniperca chuatsi*)	1% betaine/56 days	T‐AOC, SOD, CAT, and MDA =	1%	[49]
Goldfish (*Carassius auratus*)	1.4 g/kg betaine, and 2.8 g/kg betaine/42 days	Immunoenzyme activity (acid phosphatase, alkaline phosphatase, lysozyme, catalase from micrococcus lysodeikticus, total antioxidant capacity, and superoxide dismutase) 🡩	1.4 g/kg	[50]
Gibel carp (*Carassius auratus gibelio*)	0.1%, 0.5%, and 1% for betaine	Feed intake 🡩	0.5%	[51]
Gibel carp (*C. auratus gibelio*)	0.08%, 0.4% and 2% betaine/70 days	Growth performance 🡩, Lipid deposition 🡫, Lipogenic gene expression 🡫	0.4%	[52]
Gibel carp (*C. auratus gibelio*)	High‐fat diet with 1, 4, and 16 g/kg betaine/70 days	Final weight 🡫 Cholesterol synthesis, expression of HMGCR and CYP7A1 genes, intestinal lipase activity, hepatopancreas’ antioxidant capacity, and fish survival 🡩	1, 4, and 16 g/kg	[26]
Wuchang bream (*Megalobrama amblycephala*)	0.2%, 0.4%, 0.8%, and 1.6% for betaine/79 days	Feed efficiency (FW (g), SGR%, WGR%, CF%) 🡩 Liver health (the volume of hepatocytes, the number of non‐nucleated cells, and the fatty accumulation 🡫) 🡩	0.8%	[53]
Minor carp (*Labeo bata*)	0.25 g betaine in 100 g wheat flour	Growth rate % (SGR and RER) 🡩, No mortality	0.25 g betaine in 100 g wheat flour	[54]
Rohu (*Labeo rohita*)	0.25% and 0.5% Betaine/ 60 days	Growth rate % (WG, SGR, PER) 🡩 GHSR, GHRH, and IGF‐1 gene expression levels 🡩	0.25% and 0.5%	[55]
Black Seabream (*Acanthopagrus schlegelii*)	High‐fat diet (17%) supplemented with two levels (10 and 20 g/kg) of betaine	Specific growth rate and feed efficiency 🡩 Hepatic steatosis and inflammatory responses 🡫	20 g/kg	[56]
Pike perch (*Sander lucioperca*)	1%, and 2% betaine/42 days	Specific growth rate and food efficiency 🡩 Food conversion ratio 🡫	2%	[57]

## 4. The Effect of Betaine on the Fish Immune System and Its Related Mechanisms

**Figure 1:**
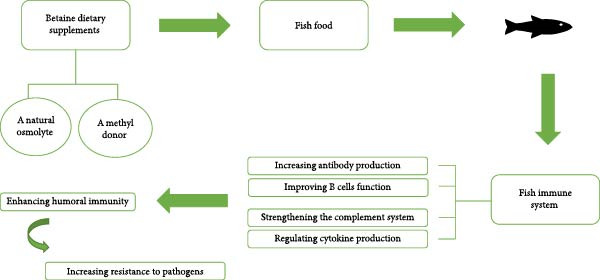
Schematic diagram summarizing the effect of betaine on the fish immune system and its related basic mechanisms.

Betaine, a natural compound with osmotic and methylating properties, has an influential function in enhancing the fish immune system (Figure 1), particularly the humoral immune response [30]. The humoral immune system includes components dissolved in the blood and other body fluids that defend against pathogens through the production of antimicrobial molecules, antibodies, and complement proteins [58–60]. Betaine enhances various mechanisms that result in the activation of appropriate humoral immune responses, thereby increasing resistance to fish pathogens.

## 5. The Effect of Betaine on Fish Humoral Immunity and Its Mechanisms

### 5.1. Antibody Production

While the direct effect of betaine on antibody titers in fish has not been well studied, investigations in other animals have demonstrated the role of this bioactive substance in increasing antibody production [61–63]. Antibodies are key proteins in the specific immune response, playing a crucial role in recognizing and neutralizing pathogens [64–66]. The mechanisms by which betaine increases antibody production can occur in several ways. As a natural osmolyte, betaine helps maintain osmotic balance and cellular stability, which can positively affect immune cells, especially lymphocytes [62]. Lymphocytes are responsible for antibody production in fish [67] and other vertebrates [68]. Nonetheless, their function can be significantly impaired under stressful conditions, such as osmotic stress. Betaine increases the survival of lymphocytes and improves their ability to produce antibodies by stabilizing the osmotic environment of immune cells.

Betaine also plays a key role in energy metabolism and can directly affect cellular function [25]. During pathogen exposure, immune cells require additional energy to mount an effective defense. Betaine increases energy levels in immune cells, particularly B cells, which are responsible for antibody production, by providing methyl groups to metabolic pathways, leading to increased B‐cell activity and antibody production [25].

In addition, betaine may directly affect B‐cell activity. After stimulation by pathogens or antigens, B cells differentiate into plasma cells, which are primarily responsible for the production and secretion of antibodies [69]. Betaine helps to improve B cells’ function and proliferation [62]. As a result, it increases the conversion of B cells into plasma cells and enhances antibody production in fish. Chronic and long‐term stresses, such as heat stress, nutritional deficiencies, or environmental pollution, can suppress the immune system and reduce antibody production in fish [70–72]. By reducing stress and decreasing the secretion of stress hormones such as cortisol [41, 73], betaine can improve immune system function and increase antibody production [62].

### 5.2. Strengthening the Complement System

The complement system, a key component of the humoral immune response of fish, consists of proteins that play a critical role in innate and specific immunity by initiating a cascade of reactions to target and eliminate pathogens [74–77]. This system functions through a series of enzymatic reactions that enhance pathogen recognition, promote phagocytosis, induce inflammation, and ultimately lead to the direct elimination of pathogens by attacking their membrane layer [74, 78–82]. Like mammals, fish have three complement activation pathways: the classical, lectin, and alternative pathways, which converge to target pathogens [79]. Recent research has reported the positive effects of dietary supplements, such as betaine, on fish immune function, including regulation of the complement system [28, 47]. As an osmolyte, betaine’s ability to stabilize proteins, including complement proteins, likely contributes to improving the activity of this system in fish [25].

Betaine appears to activate all three complement activation pathways—classical, lectin, and alternative—through its effects on immune signaling pathways. In particular, betaine’s effects on key immune‐related pathways such as mitogen‐activated protein kinase (MAPK) and nuclear factor kappa (NF‐kB), which enhance B‐cell activation, may influence the activation and function of complement proteins. These pathways are involved in inflammatory responses and immune regulation, and their modulation by betaine may enhance immune responses by the complement system.

Since betaine is also known to be a potent antioxidant [22, 29, 41, 83, 84], it may have a protective effect on tissues that produce complement proteins [85]. While the complement system is essential for immune defense, excessive or uncontrolled activation can lead to tissue damage due to inflammation. The anti‐inflammatory properties of betaine help regulate this aspect of the immune response and prevent the harmful effects of overactive complement systems [24]. By modulating the NF‐kB pathway, betaine helps control the production of inflammatory cytokines such as tumor necrosis factor‐alpha (TNF‐*α*) and interleukin‐6 (IL‐6), which are closely linked to the activation of the complement system [24, 86–88]. Specifically, betaine can reduce excessive complement activation by reducing the overproduction of inflammatory cytokines and ensure a balanced immune response that effectively neutralizes pathogens without causing tissue damage. This balancing action is crucial for maintaining immune homeostasis, especially in fish exposed to chronic stress or immune challenges.

In conclusion, several mechanisms underlie the positive effects of betaine on the fish complement system:

1. Reduction of oxidative stress: Betaine’s antioxidant properties protect complement proteins and immune cells from oxidative damage and maintain the structural integrity and functional capacity of the complement system under stress.
2. Osmotic balance: Betaine, as a natural osmolyte, helps maintain the osmotic stability of immune cells, supporting their activity and preventing dysfunction caused by osmotic stress. This osmotic regulation is critical in environments with fluctuating salinity and temperature, which are common stressors in aquaculture.
3. Regulation of signaling pathways: Betaine affects key immune signaling pathways, including MAPK and NF‐*κ*B, which are involved in the production and activation of complement proteins. By modulating these pathways, betaine helps proper activation of the complement cascade while preventing excessive inflammation.

### 5.3. Cytokine Regulation

Cytokines are vital signaling molecules that mediate and regulate immune responses in fish. They regulate interactions between immune cells, modulate inflammatory processes, and play key roles in innate and adaptive immunity [89]. Proper regulation of cytokines is essential for effective immune responses, while dysregulation can lead to chronic inflammation, immunosuppression, or immune hyperactivation.

Betaine, as a methyl donor and an osmolyte, significantly affects the regulation of cytokine production in fish [24, 83, 90]. By modulating key immune signaling pathways, betaine can help balance pro‐inflammatory and anti‐inflammatory cytokine responses and promote effective immune defense without causing excessive inflammation [24]. TNF‐α and IL‐1β are two of the most important pro‐inflammatory cytokines in fish immune responses [91–93]. These cytokines are released by immune cells such as macrophages and neutrophils after recognizing pathogens, activating inflammatory responses, and attracting other immune cells to the site of infection [94].

Studies have shown that betaine can reduce the production of TNF‐*α* and IL‐1*β* in fish under conditions of chronic inflammation or environmental stress [28, 56]. This action is particularly beneficial in preventing prolonged or excessive inflammatory responses, which can lead to tissue damage and oxidative stress. Betaine achieves this by modulating the activation of immune‐related signaling pathways such as the NF‐kB pathway, which is a key regulator of the production of pro‐inflammatory cytokines [28, 95, 96]. By reducing excessive NF‐kB activity, betaine helps to reduce the expression of genes encoding TNF‐*α*, IL‐1*β*, and other inflammatory mediators, thus preventing chronic inflammation and immune exhaustion in fish.

IL‐6 is another pro‐inflammatory cytokine that plays a dual role in fish immune responses [97, 98]. While this cytokine is crucial for initiating defense mechanisms against infections, its prolonged production can result in chronic inflammation and tissue damage. Betaine has been shown to reduce IL‐6 production by inhibiting the MAPK signaling pathway, particularly the JNK (c‐Jun N) and p38 sub‐pathways, which are associated with stress [84, 90]. By regulating IL‐6 levels, betaine not only limits excessive inflammation but also reduces oxidative stress, which is commonly associated with chronic inflammatory states in fish [28]. This action helps maintain cellular integrity and supports a more controlled immune response.

IL‐10 is a potent anti‐inflammatory cytokine that plays a principal role in limiting immune responses and preventing immune‐mediated tissue damage [99]. It suppresses the production of pro‐inflammatory cytokines and reduces the activity of immune cells after pathogen clearance [100]. Betaine has been shown to increase IL‐10 production in fish in response to high‐fat diet‐induced inflammation [56]. Betaine helps restore immune homeostasis and facilitates the resolution of inflammation by stimulating the release of IL‐10 [24].

Transforming growth factor beta (TGF‐*β*) is another key anti‐inflammatory cytokine that plays a key role in immune regulation and tissue repair. It suppresses excessive activation of the immune system and promotes the differentiation of regulatory T cells, which helps maintain immunity and prevent autoimmunity. Betaine has been observed to affect TGF‐*β* production in fish, especially during recovery from infection or stress. In the study by Lu et al. [93], betaine treatment significantly increased the mRNA expression levels of TGF‐*β* and IL‐10 in the anterior kidney and spleen of the common carp (*Plecoglossus altivelis*). This cytokine helped to resolve inflammation and promoted tissue repair and regeneration [101, 102]. Therefore, this result ensures that the fish recover more effectively from immune challenges. By upregulating TGF‐*β*, betaine contributes to a more balanced immune response and prevents the overactivation of immune cells while promoting the repair of damaged tissues.

## 6. The Effect of Betaine on Fish Cellular Immunity and Its Mechanisms

Betaine, with its unique osmotic and metabolic properties, plays an influential role in enhancing the cellular immune system [61, 103]. The cellular immune system of fish is composed of various innate immune cells, including macrophages, neutrophils, and lymphocytes, which are responsible for direct defense against pathogens [75]. Betaine enhances this aspect of fish immunity through multiple mechanisms, such as improving immune cell activity, reducing cellular stress, and regulating immune signaling pathways [30, 49, 56, 104]. These effects not only improve the overall efficiency of the fish’s immune system but also increase resistance to pathogens and reduce losses in aquaculture.

Macrophages are key players in the innate immune system, responsible for defending against pathogens and clearing damaged tissues. Phagocytosis is one of the primary functions of macrophages, allowing them to engulf and digest foreign particles, pathogens, and damaged tissues [105]. Neutrophils, a critical component of the innate immune system, also play a critical role in the frontline defense against pathogens. They fight bacterial and fungal infections through phagocytosis and the release of antimicrobial substances [106]. Research indicates that betaine can improve the phagocytic activity of macrophages and neutrophils [103, 107]. Neutrophils kill pathogens by producing antimicrobial substances, such as reactive oxygen species, nitric oxide, and enzymes such as lysozyme [108]. There is limited evidence to suggest a stimulatory action of betaine on ROS and NO production in neutrophils [103].

Macrophages regulate immune responses by producing inflammatory and anti‐inflammatory cytokines [109]. Cytokines such as TNF‐*α*, IL‐1*β*, and IL‐6 are critical for controlling inflammation and immune defense in fish [110]. Betaine affects the production of these cytokines and creates a balance between immune responses. Studies show that betaine can increase anti‐inflammatory cytokines such as IL‐10 while reducing inflammatory cytokines such as TNF‐*α* [111, 112]. This cytokine regulation improves immune balance and minimizes damage caused by inflammation [113].

Oxidative stress can damage macrophages and neutrophils and reduce their effectiveness [114–116]. The antioxidant properties of betaine can help reduce oxidative stress in cells such as immune cells [117]. Studies in fish show that betaine reduces the production of free radicals and increases the activity of antioxidant enzymes such as superoxide dismutase and catalase. This reduction in oxidative stress increases the survival and efficiency of macrophages, especially under stressful conditions or during infection.

Betaine can modulate key signaling pathways involved in macrophage and neutrophil activity, including MAPK, NF‐*κ*B, and PI3K/Akt. These pathways are essential for regulating macrophage function and cytokine production [118–120].

The NF‐kB pathway is crucial for both innate and adaptive immunity, regulating inflammation and the expression of immune‐related genes such as TNF‐*α* and IL‐1*β* [121]. Betaine modulates the NF‐kB pathway to reduce excessive inflammation and prevent cellular damage caused by chronic immune responses [24]. Under stress conditions, betaine reduces NF‐kB activity, the production of inflammatory cytokines, and long‐term inflammation [24, 122]. However, when a robust immune response is required, betaine can support the controlled activation of NF‐kB to effectively resist infections. The MAPK pathway is another key signaling pathway that controls immune cell proliferation, cytokine production, and inflammation [123]. Betaine regulates different branches of the MAPK pathway—such as ERK, JNK, and p38—to balance immune responses [122]. Activation of the ERK pathway increases lymphocyte proliferation and enhances specific immunity, while inhibition of JNK and p38 reduces inflammation and oxidative stress [124]. This dual regulation allows betaine to boost immune defenses while minimizing the harmful effects of excessive inflammation.

The phosphatidylinositol‐3‐kinase/protein kinase (PI3K/AKT) pathway is crucial in regulating immune cell survival, metabolism, and function, playing a critical role in cell proliferation and cytokine production, as well as enhancing the activity of immune cells such as macrophages and neutrophils [125]. Betaine helps support immune cell survival and function by activating the PI3K/AKT pathway [126–130].

This pathway also helps balance apoptosis (programmed cell death) and cell survival, preventing premature immune cell death and improving their efficiency in fighting pathogens. These regulatory functions are especially vital under environmental stress or chronic disease conditions, as they ensure an active and effective immune cell population.

The biochemical pathway of the target of rapamycin (mTOR) is critical for regulating immune cell growth and metabolism, particularly lymphocyte proliferation and differentiation [131]. It is also involved in protein synthesis associated with the adaptive immune response [132]. Betaine activates the mTOR pathway, thereby increasing immune cell metabolism and their ability to fight pathogens [129]. In addition, mTOR helps balance cell growth and metabolism, which is particularly important during stress and rapid immune responses [133–135]. The biochemical pathway of the activator of transcription (STAT) regulates innate and adaptive immune responses and influences the proliferation, differentiation, and immune cell functions. STAT3, in particular, is crucial for regulating anti‐inflammatory responses and preventing chronic inflammation [136, 137]. Betaine supports immune balance and directs effective responses against infection by modulating the STAT pathway while reducing the risk of tissue damage caused by excessive inflammation. In addition, STAT6 is involved in Th2 cell differentiation and the production of anti‐inflammatory cytokines. Overall, the expression and function of various STATs in fish indicate that they play a key role in modulating innate and specific immune responses [138, 139].

## 7. The Effect of Betaine on Specific Immunity and Related Mechanisms

Specific immunity, which is driven by lymphocytes, includes the production of antibodies and T cells, which are essential for disease detection and the long‐term health of fish [66]. Betaine improves the function of lymphocytes and enhances specific immune responses through various mechanisms. Lymphocytes, including B and T cells, are essential for specific immune response development and antigen recognition in fish [66]. Betaine helps to enhance the proliferation and maturation of lymphocytes by improving metabolic conditions and increasing the supply of nutrients to these cells [103, 140]. Research shows that betaine increases the number and activity of lymphocytes by optimizing the signaling pathways involved in their proliferation and maturation [122].

A key function of B lymphocytes is the production of antibodies, which have evolved to recognize and neutralize specific antigens [141]. Betaine enhances specific immune responses by stimulating B cells to produce more antibodies, such as immunoglobulin M (IgM) and immunoglobulin G (IgG) [142]. T cells are essential components in specific immunity and are responsible for recognizing and eliminating infectious or abnormal cells in fish [66]. Betaine activates signaling pathways, including PI3K/Akt and MAPK [122, 143], which play a vital role in T cell function [127]. Oxidative stress can impair immune cell function [144]. Therefore, betaine may help reduce this damage due to its potent antioxidant properties [145]. Research suggests that betaine can directly or indirectly modulate these pathways. Studies in salmon show that betaine enhances the cellular processes of macrophages and their ability to respond to pathogens by influencing the activity of the MAPK and NF‐kB pathways.

## 8. The Role of Betaine in the Antioxidant System of Fish and Its Mechanisms

Oxidative stress occurs when there is an imbalance between ROS production and the body’s antioxidant defense mechanisms. It can impair immune function and weaken the humoral immune system [146, 147]. In aquaculture, fish are exposed to various environmental stressors such as toxins, pathogens, and dietary imbalances, which lead to increased ROS production, and damaging proteins, lipids, and DNA. Fish, like other organisms, have enzymatic and non‐enzymatic antioxidant systems to neutralize free radicals and minimize oxidative damage [148, 149].

Betaine, through its methyl donor properties and osmoprotective effects, has shown great potential in enhancing the antioxidant defense of fish, improving their overall health and resistance to oxidative stress [21, 83, 84, 150]. One of the main pathways through which betaine acts is by increasing the activity of antioxidant enzymes. These enzymes include superoxide dismutase, catalase, and glutathione peroxidase, which are crucial for neutralizing ROS. Studies have shown that betaine can increase the expression and activity of these enzymes in fish, thereby reducing oxidative damage to cells [151, 152]. Other research has also indicated that betaine supplementation in fish diets increased the activity of antioxidant enzymes, reduced oxidative stress, and improved cellular protection. These properties are particularly vital during periods of environmental stress, such as temperature fluctuations, salinity changes, or pathogen exposure, which can increase ROS production [22, 129, 151, 153, 154].

In addition to enhancing enzymatic antioxidants, betaine also supports the efficacy of non‐enzymatic antioxidants such as vitamins C and E [155, 156]. These molecules are crucial in scavenging free radicals and preventing lipid peroxidation that can damage cell membranes. Betaine increases the levels and efficacy of these nonenzymatic antioxidants, further enhancing the fish’s defenses against oxidative damage. By maintaining a balance between ROS production and antioxidant defenses, betaine helps preserve the structural integrity and function of immune cells, thus supporting overall immune health in fish.

Betaine’s antioxidant properties are also linked to its ability to regulate gene expression associated with oxidative stress responses. Betaine can affect signaling pathways, such as the biochemical pathway Nrf2, which controls the expression of antioxidant proteins. By activating Nrf2, betaine helps to upregulate the transcription of various antioxidant genes, enhancing the fish’s ability to combat oxidative stress. In addition, betaine’s role in reducing levels of homocysteine, a pro‐oxidant, contributes to its antioxidant effects. Elevated homocysteine levels can exacerbate oxidative stress, and betaine helps to reduce the risk by donating a methyl group to convert homocysteine to methionine [154, 157]. This biochemical process not only protects fish from oxidative damage but also supports overall metabolic health and reduces the risk of stress‐related disorders.

## 9. The Effect of Betaine on Fish Resistance to Pathogens and Its Mechanisms

Betaine, a bioactive compound, plays a crucial role in combating aquatic pathogens. One of the primary mechanisms by which betaine combats pathogens is its regulation of cellular osmotic pressure, reducing physiological stress and enhancing host cell resistance. Additionally, betaine mitigates the severity of bacterial and viral infections by enhancing immune responses, particularly by increasing the production of anti‐inflammatory molecules and regulating immune cell activity. Some studies have shown that betaine can inhibit the growth and proliferation of pathogens and prevent the occurrence of infectious diseases in aquatic animals by affecting the metabolic pathways associated with the production of antimicrobial compounds [158–160].

In addition, betaine can influence the structure of cell membranes and increase the resistance of host cells to microbial attacks. This compound can also enhance the activity of enzymes related to cell defense and increase the production of natural antibacterial compounds in the body of aquatic animals. Some studies have shown that adding betaine to the aquatic animal’s diet can reduce the adverse effects of bacterial infections such as *Vibrio anguillarum*, *Streptococcus iniae*, *Edwardsiella tarda*, and *Aeromonas hydrophila* and increase survival rates. These properties indicate that betaine can be used as an effective dietary supplement in the management of infectious diseases in aquatic animals [22, 30, 44, 47, 93, 104, 161].

## 10. The Role of Betaine in Fish Liver Health and Related Mechanisms

The liver functions as an immunological center by producing acute phase proteins, complement proteins, and other molecules involved in innate and adaptive immune responses [162]. A healthy liver is essential for the synthesis and release of antibodies, complement factors, and other components of the humoral immune system that protect fish against pathogens [82, 163, 164]. Conversely, liver dysfunction can impair immune responses, leading to increased susceptibility to infections and reduced resistance to disease.

Oxidative stress is a principal factor that can impair liver function in fish, leading to lipid peroxidation, DNA damage, and cell apoptosis [165]. Under stressful conditions such as exposure to toxins, pollutants, or high‐density farming environments, the liver is particularly vulnerable to oxidative damage [166–170]. Betaine has been shown to exhibit potent hepatoprotective effects by reducing oxidative stress and enhancing the antioxidant defenses of the liver [84, 171]. As an osmolyte, a stabilizer of cell membranes and proteins, and as a methyl donor, it supports critical metabolic pathways such as methionine and S‐adenosylmethionine (SAMe) synthesis [25, 158, 172]. These properties help protect hepatocytes (liver cells) from oxidative damage and improve liver function. Betaine also increases the activity of key antioxidant enzymes such as superoxide dismutase, catalase, and glutathione peroxidase, which reduce ROS accumulation in the liver. By reducing oxidative stress, betaine maintains liver structure and function and supports the liver’s capacity to contribute to the immune system, including immune‐related protein productions. In fish, the liver is the primary site of lipid metabolism, where fatty acids are synthesized, stored, and transported [173].

Disturbances in lipid metabolism, often due to poor diet or stress, can lead to fatty liver disease, which compromises liver function and immune capacity in fish [174, 175]. Betaine has been shown to regulate lipid metabolism in fish by stimulating the conversion of homocysteine to methionine, which supports phosphatidylcholine synthesis, a key component of cell membranes and lipoproteins [176]. This action helps maintain lipid homeostasis in the liver, reducing the risk of fat accumulation and promoting overall liver health. A healthy liver, as a vital organ in immunity, can help fish withstand challenging environmental stressors [177].

Fish liver is responsible for detoxifying harmful substances and synthesizing proteins essential for immune function, such as immunoglobulins (antibodies) and complement proteins [178–180]. Betaine supports liver detoxification processes by increasing the production of SOD, GPx, CAT, and glutathione, the principal antioxidants that neutralize toxic substances. This detoxifying effect prevents liver cell damage and enhances the liver’s ability to maintain its role in immune function. In addition, betaine’s role as a methyl donor aids protein synthesis by providing the necessary methyl groups for the production of SAMe, which is involved in DNA, RNA, and protein methylation [21, 181]. This process ensures that the liver can effectively synthesize immune‐related proteins, supporting liver health and humoral immunity.

## 11. Conclusion

Overall, this study summarized the effect of betaine dietary supplements on the fish immune system and related mechanisms. Betaine plays a significant role in regulating cellular processes due to its biochemical and physiological properties. Moreover, it helps fish cope with environmental stress by maintaining osmotic balance and regulating water and electrolytes in cells, especially in salinity and temperature fluctuations. Furthermore, this study highlights that betaine enhances the innate defense mechanisms of fish against pathogens by increasing the activity of immune cells, especially macrophages, regulating cytokine production, and reducing inflammation. In addition, betaine’s antioxidant properties reduce oxidative stress and improve immune signaling, helping fish maintain optimal immune function even in stressful environments. Due to these immunogenic properties, it has become a suitable and safe additive for aquaculture that can be, to some extent, a potentially effective alternative to antibiotics and chemicals. While dietary betaine has shown promising effects on growth, immunity, and antioxidant status in various fish species, its application in aquaculture still presents several challenges. Potential risks such as species‐specific tolerance, dose‐dependent adverse effects, and long‐term safety remain underexplored. In addition, despite its potential as a feed additive, there are notable knowledge gaps that must be addressed. Future studies should investigate the interaction of betaine with other nutritional supplements to evaluate synergistic effects on growth, metabolism, and health of fish; investigating the effect of betaine on different stages of fish development (larvae, juveniles, adults) to determine stage‐specific sensitivities and different physiological responses; evaluation of the long‐term effects of betaine consumption on general health, reproductive performance, and fish meat quality under industrial farming conditions; and studying the effects of betaine on the expression of genes related to immunity and oxidative stress using genomics and transcriptomics techniques.

## Ethics Statement

Not any animals were used in the present research.

## Consent

The authors have nothing to report.

## Disclosure

After using the tool (ChatGPT‐3.5), the authors reviewed and edited the content as needed and take full responsibility for the content of the publication.

## Conflicts of Interest

The authors declare no conflicts of interest.

## Author Contributions

Morteza Yousefi was responsible for supervision, funding acquisition, resources, and writing – review and editing. Taravat Molayemraftar was responsible for writing the early draft. Seyyed Morteza Hoseini was responsible for conceptualization and review of the draft. Hamed Ghafarifarsani was responsible for writing – review and editing.

## Funding

There is no government or organizational fund for this work.

## Data Availability

All data generated or analyzed during this study were included in this published article.

## References

[1] Verdegem M. , Buschmann A. H. , Latt U. W. , Dalsgaard A. J. T. , and Lovatelli A. , The Contribution of Aquaculture Systems to Global Aquaculture Production, Journal of the World Aquaculture Society. (2023) 54, no. 2, 206–250, 10.1111/jwas.12963.

[2] Ahmad A. , Abdullah S. R. S. , Hasan H. A. , Othman A. R. , and Ismail N. I. , Aquaculture Industry: Supply and Demand, Best Practices, Effluent and Its Current Issues and Treatment Technology, Journal of Environmental Management. (2021) 287, 10.1016/j.jenvman.2021.112271, 112271. 33706093

[3] Ciji A. and Akhtar M. S. , Stress Management in Aquaculture: A Review of Dietary Interventions, Reviews in Aquaculture. (2021) 13, no. 4, 2190–2247, 10.1111/raq.12565.

[4] Van Doan H. , Prakash P. , and Hoseinifar S. H. , et al.Marine-Derived Products as Functional Feed Additives in Aquaculture: A Review, Aquaculture Reports. (2023) 31, 10.1016/j.aqrep.2023.101679, 101679.

[5] Lim L. S. , Ebi S. , and Tan K. , et al. Faudzi N. M. , Shah M. D. , Mazlan N. , and Shaleh S. R. M. , Feeding Stimulants in Finfish Aquaculture, Essentials of Aquaculture Practices, 2024, Springer, 10.1007/978-981-97-6699-4_5.

[6] Li M.-Y. , Shi Y.-C. , Xu W.-X. , Zhao L. , and Zhang A.-Z. , Exploring Cr(VI)-Induced Blood-Brain Barrier Injury and Neurotoxicity in Zebrafish and Snakehead Fish, and Inhibiting Toxic Effects of Astaxanthin, Environmental Pollution. (2024) 355, 10.1016/j.envpol.2024.124280, 124280. 38815890

[7] Zhao L. , Zhang Y. , and Yu J. , et al.Astaxanthin Ameliorates Cr(VI) Accumulation, Antioxidant, Digestive, Apoptosis and Inflammatory Response, Regional Studies in Marine Science. (2025) 87, 10.1016/j.rsma.2025.104240, 104240.

[8] Yu Z. , Xiong Z. , and Wang Y. , et al.Exploring *Allium mongolicum* Regel Flavonoids Alleviation of Deoxynivalenol-Induced Gut Inflammation and Flesh-Quality Improvement Based on Muscle-Intestinal Axis in a *Channa argus* Model, Food Bioscience. (2025) 71, 10.1016/j.fbio.2025.107187, 107187.

[9] Banaee M. , Sharma D. , Sinha R. , Mikušková N. , Velíšek J. , and Faggio C. , Herbal Remedies in Aquaculture: Efficacy, Risks, and the Need for Quality Assurance, Aquaculture International. (2025) 33, no. 6, 10.1007/s10499-025-02120-7, 492.

[10] Du J.-H. , Xu M.-Y. , Wang Y. , Lei Z. , Yu Z. , and Li M.-Y. , Evaluation of *Taraxacum mongolicum* Flavonoids in Diets for *Channa argus* Based on Growth Performance, Immune Responses, Apoptosis and Antioxidant Defense System Under Lipopolysaccharide Stress, Fish & Shellfish Immunology. (2022) 131, 1224–1233, 10.1016/j.fsi.2022.11.034. 36414130

[11] Niu X.-T. , Sun C. , Zhao L. , Chen X.-M. , Wang G.-Q. , and Li M.-Y. , The Major Role of Glucocorticoid Receptor (GR) in Astaxanthin Alleviates Immune Stress in *Channa argus* Lymphocyte, Aquaculture. (2024) 584, 10.1016/j.aquaculture.2024.740637, 740637.

[12] Demeke A. and Tassew A. , A Review on Water Quality and Its Impact on Fish Health, International Journal of Fauna and Biological Studies. (2016) 3, no. 1, 21–31.

[13] Lulijwa R. , Rupia E. J. , and Alfaro A. C. , Antibiotic use in Aquaculture, Policies and Regulation, Health and Environmental Risks: A Review of the top 15 Major Producers, Reviews in Aquaculture. (2020) 12, no. 2, 640–663, 10.1111/raq.12344, 2-s2.0-85064003616.

[14] Jijie R. , Mihalache G. , and Balmus I. M. , et al.Zebrafish as a Screening Model to Study the Single and Joint Effects of Antibiotics, Pharmaceuticals. (2021) 14, no. 6, 10.3390/ph14060578, 578. 34204339 PMC8234794

[15] Bondad-Reantaso M. G. , MacKinnon B. , and Karunasagar I. , et al.Review of Alternatives to Antibiotic use in Aquaculture, Reviews in Aquaculture. (2023) 15, no. 4, 1421–1451, 10.1111/raq.12786.

[16] Rashidian G. , Bahrami Gorji S. , Farsani M. N. , Prokić M. D. , and Faggio C. , The Oak (*Quercus brantii*) Acorn as a Growth Promotor for Rainbow Trout (*Oncorhynchus mykiss*): Growth Performance, Body Composition, Liver Enzymes Activity and Blood Biochemical Parameters, Natural Product Research. (2020) 34, no. 17, 2413–2423, 10.1080/14786419.2018.1538994, 2-s2.0-85059082078. 30580593

[17] Rashidian G. , Zare M. , Tabibi H. , Stejskal V. , and Faggio C. , The Synergistic Effects of Four Medicinal Plant Seeds and Chelated Minerals on the Growth, Immunity, and Antioxidant Capacity of Rainbow Trout (*Oncorhynchus mykiss*), Fish & Shellfish Immunology. (2023) 139, 10.1016/j.fsi.2023.108930, 108930. 37419436

[18] Pastaki N. J. , Abdollahpour H. , Karimzadeh M. , Zamani H. , Multisanti C. R. , and Faggio C. , Physiological and Immunological Impact of Lavender (*Lavandula angustifolia*) on Female Goldfish (*Carassius auratus*), Aquaculture Reports. (2023) 33, no. 3, 10.1016/j.aqrep.2023.101841, 101841.

[19] Li M.-Y. , Liu Y.-Z. , and Chen X.-M. , et al.Astaxanthin Ameliorates High-Carbohydrate Diet-Induced ER Stress, Immunosuppression and Hepatic Glucose Metabolism Through AMPK/Autophagy Pathway in *Channa argus* , Aquaculture. (2025) 598, 10.1016/j.aquaculture.2024.742010, 742010.

[20] Dong X. , Wang J. , and Ji P. , et al.Dietary Betaine Supplementation Promotes Growth, n-3 LC-PUFA Content and Innate Immunity in *Macrobrachium rosenbergii* , Aquaculture. (2020) 525, 10.1016/j.aquaculture.2020.735308, 735308.

[21] Arumugam M. K. , Paal M. C. , Donohue T. M.Jr., Ganesan M. , Osna N. A. , and Kharbanda K. K. , Beneficial Effects of Betaine: A Comprehensive Review, Biology. (2021) 10, no. 6, 10.3390/biology10060456, 456. 34067313 PMC8224793

[22] Mugwanya M. , Dawood M. A. O. , Kimera F. , and Sewilam H. , A Meta-Analysis on the Influence of Dietary Betaine on the Growth Performance and Feed Utilization in Aquatic Animals, Aquaculture Reports. (2024) 37, 10.1016/j.aqrep.2024.102200, 102200.

[23] Dobrijević D. , Pastor K. , and Nastić N. , et al.Betaine as a Functional Ingredient: Metabolism, Health-Promoting Attributes, Food Sources, Applications and Analysis Methods, Molecules. (2023) 28, no. 12, 10.3390/molecules28124824, 4824. 37375378 PMC10302777

[24] Zhao G. , He F. , and Wu C. , et al.Betaine in Inflammation: Mechanistic Aspects and Applications, Frontiers in Immunology. (2018) 9, 10.3389/fimmu.2018.01070, 2-s2.0-85047464860, 1070. 29881379 PMC5976740

[25] Figueroa-Soto C. G. and Valenzuela-Soto E. M. , Glycine Betaine Rather Than Acting Only as an Osmolyte Also Plays a Role as Regulator in Cellular Metabolism, Biochimie. (2018) 147, 89–97, 10.1016/j.biochi.2018.01.002, 2-s2.0-85041410129. 29366935

[26] Dong X. , Wang J. , and Zhao M. , et al.Betaine Alleviates High-Fat Diet-Induced Excessive Lipid Deposition in Gibel Carp Hepatopancreas and L8824 Cells by Enhancing VLDL Secretion Through HNF4*α*/MTTP Pathway, Aquaculture Nutrition. (2024) 2024, 10.1155/2024/8886237, 8886237. 38469394 PMC10927341

[27] Buonaiuto G. , Federiconi A. , Vecchiato C. G. , Benini E. , and Mordenti A. L. , Betaine Dietary Supplementation: Healthy Aspects in Human and Animal Nutrition, Antioxidants. (2025) 14, no. 7, 10.3390/antiox14070771, 771. 40722875 PMC12291949

[28] Sun H. , Jiang W. D. , and Wu P. , et al.Betaine Supplementations Enhance the Intestinal Immunity of on-Growing Grass Carp (*Ctenopharyngodon idella*): Partly Related to TOR and NF-*κ*B Signaling Pathways, Aquaculture. (2020) 518, 10.1016/j.aquaculture.2019.734846, 734846.

[29] Mohseni M. , Saltanat N. L. , Rastravan M. E. , and Golalipour Y. , Effects of Betaine Supplementation in Plant-Protein-Based Diets on Growth Performance, Haemato-Immunological Parameters, Antioxidant Status and Digestive Enzyme Activities of Juvenile Caspian Trout (*Salmo trutta*, Kessler, 1877), Aquaculture Nutrition. (2021) 27, no. 6, 2132–2141, 10.1111/anu.13348.

[30] Choi H. J. , Lee J.-H. , and Choi D. Y. , et al.Innate Immunity and Protective Effects of Orally Administered Betaine Against Viral and Bacterial Diseases in the Olive Flounder *Paralichthys olivaceus* (Temminck & Schlegel), Journal of Fish Diseases. (2022) 45, no. 11, 1789–1798, 10.1111/jfd.13700. 35934929

[31] Naresh Chary V. , Dinesh Kumar C. , Vairamani M. , and Prabhakar S. , Characterization of Amino Acid-Derived Betaines by Electrospray Ionization Tandem Mass Spectrometry, Journal of Mass Spectrometry. (2012) 47, no. 1, 79–88, 10.1002/jms.2029, 2-s2.0-84856352393. 22282093

[32] Kheder A. M. A. and Abo-Elmagd A. M. , Improving Vegetative Growth and Productivity of Navel Orange (*Citrus sinensis* L.) Trees under Salt Affected Soil Using Glycinebetaine and Potassium Silicate, Journal of Plant Production. (2021) 12, no. 3, 279–286, 10.21608/jpp.2021.158995.

[33] Finkelstein J. D. , Harris B. J. , Martin J. J. , and Kyle W. E. , Regulation of Hepatic Betaine-Homocysteine Methyltransferase by Dietary Methionine, Biochemical and Biophysical Research Communications. (1982) 108, no. 1, 344–348, 10.1016/0006-291X(82)91872-1, 2-s2.0-0020485237. 7150291

[34] Takis P. G. , Papavasileiou K. D. , Troganis A. N. , and Melissas V. S. , The Chemistry of Betaine, Betaine: Chemistry, Analysis, Function and Effects, 2015, Royal Society of Chemistry.

[35] Lever M. and Slow S. , The Clinical Significance of Betaine, An Osmolyte With a Key Role in Methyl Group Metabolism, Clinical Biochemistry. (2010) 43, no. 9, 732–744, 10.1016/j.clinbiochem.2010.03.009, 2-s2.0-77952951396. 20346934

[36] Eklund M. , Bauer E. , Wamatu J. , and Mosenthin R. , Potential Nutritional and Physiological Functions of Betaine in Livestock, Animal Science. (2005) 81, no. 1, 3–11. 10.1079/NRR200493 19079893

[37] Luo D. , Wang X. , Cao S. , Ba L. , and Chen J. , Recent Advances in Betaine: Extraction, Identification, and Bioactive Functions in Human Health, Food Science & Nutrition. (2025) 13, no. 4, 10.1002/fsn3.70173, e70173. 40255548 PMC12006033

[38] dos Santos Sanchez M. S. , Rodrigues M. L. , Pessini J. E. , Bittencourt F. , Boscolo W. R. , and Signor A. , Assessment of the Relative Bioavailability Between Betaine and l-Carnitine for Nile Tilapia, Aquaculture International. (2025) 33, 10.1007/s10499-024-01771-2, 80.

[39] dos S. Sanchez M. S. , Lins-Rodrigues S. , and Pessini M. , et al.Dietary Supplementation of Betaine Improves Growth Performance and Reduces Lipid Peroxidation in Nile Tilapia, Aquaculture Nutrition. (2021) 27, no. 6, 1861–1870, 10.1111/anu.13323.

[40] Risha E. , Ahmed F. , Khaled A. A. , Hossain F. M. A. , Akhtar N. , and Zahran E. , Interactive Effects of Dietary Betaine and Chromium Picolinate on the Immunomodulation, Antioxidative Response and Disease Resistance of Nile Tilapia (*Oreochromis niloticus*), Aquaculture Research. (2022) 53, no. 9, 3464–3477, 10.1111/are.15853.

[41] Adjoumani J.-J. Y. , Wang K. , Zhou M. , Liu W. , and Zhang D. , Effect of Dietary Betaine on Growth Performance, Antioxidant Capacity and Lipid Metabolism in Blunt Snout Bream Fed a High-Fat Diet, Fish Physiology and Biochemistry. (2017) 43, no. 6, 1733–1745, 10.1007/s10695-017-0405-9, 2-s2.0-85030164792. 28963592

[42] Yeşilayer N. and Kaymak I. E. , Effect of Partial Replacement of Dietary Fish Meal by Soybean Meal With Betaine Attractant Supplementation on Growth Performance and Fatty Acid Profiles of Juvenile Rainbow Trout (*Oncorhynchus mykiss*), Aquaculture Research. (2020) 51, no. 4, 1533–1541, 10.1111/are.14501.

[43] Pinedo-Gil J. , Martín-Diana A. B. , Bertotto D. , Sanz-Calvo M.Á. , Jover-Cerdá M. , and Tomás-Vidal A. , Effects of Dietary Inclusions of Red Beet and Betaine on the Acute Stress Response and Muscle Lipid Peroxidation in Rainbow Trout, Fish Physiology and Biochemistry. (2018) 44, no. 3, 939–948, 10.1007/s10695-018-0483-3, 2-s2.0-85047181405. 29511985

[44] Awad L. Z. , El-Mahallawy H. S. , Abdelnaeim N. S. , Mahmoud M. M. A. , Dessouki A. A. , and ElBanna N. I. , Role of Dietary Spirulina Platensis and Betaine Supplementation on Growth, Hematological, Serum Biochemical Parameters, Antioxidant Status, Immune Responses, and Disease Resistance in Nile Tilapia, Fish & Shellfish Immunology. (2022) 126, 122–130, 10.1016/j.fsi.2022.05.040. 35613669

[45] Luo Z. , Tan X.-Y. , Liu X.-J. , and Wen H. , Effect of Dietary Betaine Levels on Growth Performance and Hepatic Intermediary Metabolism of GIFT Strain of Nile Tilapia Orechromis Niloticus Reared İn Freshwater, Aquaculture Nutrition. (2011) 17, no. 4, 361–367, 10.1111/j.1365-2095.2010.00805.x, 2-s2.0-79960123826.

[46] Murthy H S. , Akshaya M. , and Prakash P. , Effect of Betaine Hydrochloride as Feed Attractant on Growth, Survival and Feed Utilization of Common Carp, *Cyprinus carpio* , Journal of Aquaculture & Marine Biology. (2016) 4, no. 3, 83–90, 10.15406/jamb.2016.04.00083.

[47] Hajirezaee S. , Ramezani S. , and Ahani S. , Betaine and the Probiotic, *Lactobacillus rhamnosus* in the Diet of the Common Carp, *Cyprinus carpio*: Effects on Growth, Digestive Enzyme Activities, Antioxidant System, Humoral and Mucosal Immunity and Resistance to *Streptococcus iniae* , Aquaculture Reports. (2024) 38, 10.1016/j.aqrep.2024.102282, 102282.

[48] Ali H. H. A. and Al-faragi J. K. A. , Efficiency of Betaine and *β* -Glucan as Feed Additives on the Growth Performance and Survival Rate of Common Carp (*Cyprinus carpio* L.) Fingerlings, Journal of Endocrinology. (2017) 5, 27–31.

[49] Li H. , Zeng Y. , and Zheng X. , et al.Dietary Betaine Attenuates High-Carbohydrate-Diet-Induced Oxidative Stress, Endoplasmic Reticulum Stress, and Apoptosis in Mandarin Fish (*Siniperca chuatsi*), Antioxidants. (2023) 12, no. 10, 10.3390/antiox12101860, 1860. 37891939 PMC10604392

[50] Shi L. , Wei L. , Zhai H. , Ren T. , and Han Y. , An Evaluation on Betaine and Trimethylammonium Hydrochloride in the Diet of *Carassius auratus*: Growth, Immunity, and Fat Metabolism Gene Expression, Aquaculture Reports. (2021) 19, 10.1016/j.aqrep.2021.100627, 100627.

[51] Xue M. and Cui Y. , Effect of Several Feeding Stimulants on Diet Preference by Juvenile Gibel Carp (*Carassius auratus gibelio*), Fed Diets With or Without Partial Replacement of Fish Meal by Meat and Bone Meal, Aquaculture. (2001) 198, no. 3-4, 281–292, 10.1016/S0044-8486(00)00602-5, 2-s2.0-0035796810.

[52] Dong X. , Xue W. , and Hua J. , et al.Effects of Dietary Betaine in Allogynogenetic Gibel Carp (*Carassius auratus gibelio*): Enhanced Growth, Reduced Lipid Deposition and Depressed Lipogenic Gene Expression, Aquaculture Research. (2018) 49, no. 5, 1967–1972, 10.1111/are.13652, 2-s2.0-85043227178.

[53] Pan W. , Wang F. , and Xu J. , et al.Betaine Supplementation Into High-Carbohydrate Diets Improves Feed Efficiency and Liver Health of *Megalobrama amblycephala* by Increasing Taurine Synthesis, Aquaculture Nutrition. (2024) 2024, 10.1155/2024/9632883, 9632883. 39555516 PMC11469934

[54] Ghosh T. K. , Chauhan Y. H. , and Mandal R. N. , Growth Performance of *Labeo bata* (Hamilton, 1822) in Freshwater and Its Acclimatization in Brackish Water With Betaine as Feed Additive, Aquaculture. (2019) 501, 128–134, 10.1016/j.aquaculture.2018.11.020, 2-s2.0-85056478705.

[55] Dar S. A. , Srivastava P. P. , and Varghese T. , et al.Expression of Growth and Hunger Related Genes and Physio-Biochemical Responses in *Labeo rohita* (Hamilton, 1822) Fed With Lysine and Betaine, Cellular Physiology and Biochemistry. (2019) 53, no. 5, 851–864, 10.33594/000000177. 31714043

[56] Jin M. , Shen Y. , and Pan T. , et al.Dietary Betaine Mitigates Hepatic Steatosis and Inflammation Induced by a High-Fat-Diet by Modulating the Sirt1/Srebp-1/Pparɑ Pathway in Juvenile Black Seabream (*Acanthopagrus schlegelii*), Frontiers in Immunology. (2021) 12, 10.3389/fimmu.2021.694720, 694720. 34248992 PMC8261298

[57] Zakipour R. E. , Akbari M. , Arshadi A. , and Effatpanah E. , Effect of Different Levels of Dietary Betaine on Growth Performance, Food Efficiency and Survival Rate of Pike Perch (*Sander lucioperca*) Fingerlings, Iranian Journal of Fisheries Sciences. (2012) 11, 902–910.

[58] Muiswinkel W. V. and Vervoorn-Van Der Wal B. , The Immune System of Fish, Fish Diseases and Disorders. Volume 1: Protozoan and Metazoan Infections, 2006, CABI, 678–701.

[59] Whyte S. K. , The Innate Immune Response of Finfish—A Review of Current Knowledge, Fish & Shellfish Immunology. (2007) 23, no. 6, 1127–1151, 10.1016/j.fsi.2007.06.005, 2-s2.0-35648988449. 17980622

[60] Mustafa E. and AL-Taee S. , Innate and Adaptive Immunity in Fish: A Review, Al-Anbar Journal of Veterinary Sciences. (2020) 13, no. 2, 10.37940/AJVS.2020.13.2.1.

[61] Chand N. , Naz S. , Maris H. , Khan R. U. , Khan S. , and Qureshi M. S. , Effect of Betaine Supplementation on the Performance and Immune Response of Heat Stressed Broilers, Pakistan Journal of Zoology. (2017) 49, no. 5, 1857–1862, 10.17582/journal.pjz/2017.49.5.1857.1862, 2-s2.0-85031727399.

[62] Kumar T. , Yadav M. , and Singh L. R. , Role of Osmolytes in Regulating Immune System, Current Pharmaceutical Design. (2016) 22, no. 20, 3050–3057, 10.2174/1381612822666160307150059, 2-s2.0-84973595036. 26951102

[63] Ratriyanto A. and Mosenthin R. , Osmoregulatory Function of Betaine in Alleviating Heat Stress in Poultry, Journal of Animal Physiology and Animal Nutrition. (2018) 102, no. 6, 1634–1650, 10.1111/jpn.12990, 2-s2.0-85053503325. 30238641

[64] Morrison R. N. and Nowak B. F. , The Antibody Response of Teleost Fish, Seminars in Avian and Exotic Pet Medicine, 2002, WB Saunders, 46–54.

[65] Firdaus-Nawi M. and Zamri-Saad M. , Major Components of Fish Immunity: A Review, Pertanika Journal of Tropical Agricultural Science. (2016) 39, no. 4.

[66] Kordon A. O. , Pinchuk L. , and Karsi A. , Adaptive Immune System in Fish, Turkish Journal of Fisheries and Aquatic Sciences. (2021) 22, no. 4, 10.4194/TRJFAS20235, TRJFAS20235.

[67] Scapigliati G. , Functional Aspects of Fish Lymphocytes, Developmental & Comparative Immunology. (2013) 41, no. 2, 200–208, 10.1016/j.dci.2013.05.012, 2-s2.0-84880167760. 23707785

[68] Orakpoghenor O. , Avazi D. O. , Markus T. P. , and Olaolu O. S. , Lymphocytes: A Brief Review, SCIRES Literature. (2019) 3, no. 1, 5–8.

[69] Akkaya M. , Kwak K. , and Pierce S. K. , B Cell Memory: Building Two Walls of Protection Against Pathogens, Nature Reviews Immunology. (2020) 20, no. 4, 229–238, 10.1038/s41577-019-0244-2. PMC7223087 31836872

[70] Pulsford A. L. , Lemaire-Gony S. , and Farley S. , Effects of Stress on the Immune System of Fish, 1994.

[71] Einarsdóttir I. E. , Nilssen K. J. , and Iversen M. , Effects of Rearing Stress on Atlantic Salmon (*Salmo salar* L.) Antibody Response to a Non-Pathogenic Antigen, Aquaculture Research. (2000) 31, no. 12, 923–930, 10.1046/j.1365-2109.2000.00506.x, 2-s2.0-0034545384.

[72] Tort L. , Stress and Immune Modulation in Fish, Developmental & Comparative Immunology. (2011) 35, no. 12, 1366–1375, 10.1016/j.dci.2011.07.002, 2-s2.0-83355166840. 21782845

[73] Varghese T. , SanalEbeneeza S. A. D. , and Pal A. K. , Mitigation of Stress in Fish Through Nutraceuticals, Development. (2021) 3, no. 1, 1014.

[74] Boshra H. , Li J. , and Sunyer J. O. , Recent Advances on the Complement System of Teleost Fish, Fish & Shellfish Immunology. (2006) 20, no. 2, 239–262, 10.1016/j.fsi.2005.04.004, 2-s2.0-22844451392. 15950490

[75] Mokhtar D. M. , Zaccone G. , Alesci A. , Kuciel M. , Hussein M. T. , and Sayed R. K. , Main Components of Fish Immunity: An Overview of the Fish Immune System, Fishes. (2023) 8, no. 2, 10.3390/fishes8020093, 93.

[76] Guha R. , Beegum S. , and Kumar N. , et al. Das A. , Ghosh S. , and Nayak A. K. , Fish Immune Response: An Overview, Bacillus Probiotics for Sustainable Aquaculture, 2024, 1st edition, CRC Press, 10.1201/9781003503811-2.

[77] Awad E. , The Role of Natural Products on the Immune Status of Freshwater Fish, Aquaculture International. (2025) 33, no. 6, 10.1007/s10499-025-02035-3, 396.

[78] Yu Z. , Xu S.-F. , Zhao J.-L. , Zhao L. , Zhang A.-Z. , and Li M.-Y. , Toxic Effects of Hexavalent Chromium (Cr^6+^) on Bioaccumulation, Apoptosis, Oxidative Damage and Inflammatory Response in *Channa asiatica* , Environmental Toxicology and Pharmacology. (2021) 87, 10.1016/j.etap.2021.103725, 103725. 34416396

[79] Bavia L. , Santiesteban-Lores L. E. , Carneiro M. C. , and Prodocimo M. M. , Advances in the Complement System of a Teleost Fish, *Oreochromis niloticus* , Fish & Shellfish Immunology. (2022) 123, 61–74, 10.1016/j.fsi.2022.02.013. 35227880

[80] Yu Z. , Zhao L. , and Zhao J.-L. , et al.Dietary *Taraxacum mongolicum* Polysaccharide Ameliorates the Growth, Immune Response, and Antioxidant Status in Association With NF-*κ*B, Nrf2 and TOR in Jian Carp (*Cyprinus carpio* var. Jian), Aquaculture. (2022) 547, 10.1016/j.aquaculture.2021.737522, 737522.

[81] Jeyavani J. , Sibiya A. , Sivakamavalli J. , Elumalai P. , Baskaralingam V. , and Faggio C. , Phytotherapy and Combined Nanoformulations as a Promising Disease Management in Aquaculture: A Review, Aquaculture International. (2022) 30, no. 2, 1071–1086, 10.1007/s10499-022-00848-0.

[82] Shadmand M. , Gholamhosseini A. , and Yektaseresht A. , et al.Investigation of the Oral Effects of Alcoholic Extract of Wild Yarrow (*Achillea wilhelmsii*) on Growth Performance, Immune, and Biochemical Serum Responses in Rainbow Trout (*Oncorhynchus mykiss*), Aquaculture Nutrition. (2025) 2025, 10.1155/anu/2360780, 2360780. 40230616 PMC11996263

[83] Kaur S. , Sharma N. , and Vyas M. , et al.A Review on Pharmacological Activities of Betaine, Plant Archives. (2019) 19, 1021–1034.

[84] Wang C. , Ma C. , Gong L. , Dai S. , and Li Y. , Preventive and Therapeutic Role of Betaine in Liver Disease: A Review on Molecular Mechanisms, European Journal of Pharmacology. (2021) 912, 10.1016/j.ejphar.2021.174604, 174604. 34743980

[85] Nakao M. , Tsujikura M. , Ichiki S. , Vo T. K. , and Somamoto T. , The Complement System in Teleost Fish: Progress of Post-Homolog-Hunting Researches, Developmental & Comparative Immunology. (2011) 35, no. 12, 1296–1308, 10.1016/j.dci.2011.03.003, 2-s2.0-83355169786. 21414344

[86] Hagar H. , El Medany A. , Salam R. , El Medany G. , and Nayal O. A. , Betaine Supplementation Mitigates Cisplatin-Induced Nephrotoxicity by Abrogation of Oxidative/Nitrosative Stress and Suppression of Inflammation and Apoptosis in Rats, Experimental and Toxicologic Pathology. (2015) 67, no. 2, 133–141, 10.1016/j.etp.2014.11.001, 2-s2.0-84977784322. 25488130

[87] Yang J.-M. , Zhou R. , Zhang M. , Tan H.-R. , and Yu J.-Q. , Betaine Attenuates Monocrotaline-Induced Pulmonary Arterial Hypertension in Rats via Inhibiting Inflammatory Response, Molecules. (2018) 23, no. 6, 10.3390/molecules23061274, 2-s2.0-85047870808, 1274. 29861433 PMC6100216

[88] Jorgačević B. , Stanković S. , Filipović J. , Samardžić J. , Vučević D. , and Radosavljević T. , Betaine Modulating MIF-Mediated Oxidative Stress, Inflammation and Fibrogenesis in Thioacetamide-Induced Nephrotoxicity, Current Medicinal Chemistry. (2022) 29, no. 31, 5254–5267, 10.2174/0929867329666220408102856. 35400322

[89] Reyes-Cerpa S. , Maisey K. , Reyes-López F. , Toro-Ascuy D. , Sandino A. M. , and Imarai M. , Fish Cytokines and Immune Response, New Advances and Contributions to Fish Biology, 2012, 1, IntechOpen, 10.5772/53504.

[90] Xia Y. , Chen S. , Zhu G. , Huang R. , Yin Y. , and Ren W. , Betaine Inhibits Interleukin-1*β* Production and Release: Potential Mechanisms, Frontiers in Immunology. (2018) 9, 10.3389/fimmu.2018.02670, 2-s2.0-85057397355, 2670. 30515160 PMC6255979

[91] Sigh J. , Lindenstrøm T. , and Buchmann K. , Expression of Pro-Inflammatory Cytokines in Rainbow Trout (*Oncorhynchus mykiss*) During an Infection With *Ichthyophthirius multifiliis* , Fish & Shellfish Immunology. (2004) 17, no. 1, 75–86, 10.1016/j.fsi.2003.12.005, 2-s2.0-3042545384. 15145419

[92] Yildirim N. C. and Danabas S. , Assessment of Immunomodulator Biomarkers (Tnf-*α*, Il-1*β* and Il-6) in Liver of *Capoeta umbla* for Biomonitoring of Pollution in Uzuncayir Dam Lake (Tunceli, Turkey), IJFS. (2014) 13, no. 3, 653–666.

[93] Lu J.-F. , Luo S. , and Jin T.-C. , et al.Betaine Protects Ayu (*Plecoglossus altivelis*) Against Vibrio Anguillarum Infection in Salinity by Regulating the Immunomodulatory Activity of Monocytes/Macrophages, Aquaculture. (2021) 536, 10.1016/j.aquaculture.2021.736482, 736482.

[94] Michlewska S. , Macrophage Phagocytosis of Apoptotic Neutrophils Is Critically Regulated by the Opposing Actions of Pro-Inflammatory and Anti-Inflammatory Agents: Key Role for TNF-*α* , The FASEB Journal. (2011) 23, 844–854. 10.1096/fj.08-121228 18971259

[95] Habte-Tsion H.-M. , A Review on Fish Immuno-Nutritional Response to Indispensable Amino Acids in Relation to TOR, NF-*κ*B and Nrf2 Signaling Pathways: Trends and Prospects, Comparative Biochemistry and Physiology Part B: Biochemistry and Molecular Biology. (2020) 241, 10.1016/j.cbpb.2019.110389, 110389. 31812790

[96] Yan X. , Zhao X. , Huo R. , and Xu T. , IRF3 and IRF8 Regulate NF-*κ*B Signaling by Targeting MyD88 in Teleost Fish, Frontiers in Immunology. (2020) 11, 10.3389/fimmu.2020.00606, 606. 32373114 PMC7179762

[97] Castellana B. , Iliev D. B. , and Sepulcre M. P. , et al.Molecular Characterization of Interleukin-6 in the Gilthead Seabream (*Sparus aurata*), Molecular Immunology. (2008) 45, no. 12, 3363–3370, 10.1016/j.molimm.2008.04.012, 2-s2.0-44749084338. 18513800

[98] Wang X. , Chen J. , Zhang R. , Liu L. , Ma G. , and Zhu H. , Interleukin-6 in Siberian Sturgeon (*Acipenser Baeri*): Molecular Characterization and Immune Functional Activity, Fish & Shellfish Immunology. (2020) 102, 296–306, 10.1016/j.fsi.2020.03.023. 32184192

[99] Zhou X. , Schmidtke P. , Zepp F. , and Meyer C. U. , Boosting Interleukin-10 Production: Therapeutic Effects and Mechanisms, Current Drug Targets — Immune, Endocrine & Metabolic Disorders. (2005) 5, no. 4, 465–475, 10.2174/156800805774912926, 2-s2.0-28444449495. 16375698

[100] Howes A. , Stimpson P. , Redford P. , Gabrysova L. , and O’Garra A. , Interleukin-10: Cytokines in Anti-Inflammation and Tolerance, Cytokine Frontiers: Regulation of Immune Responses in Health and Disease, 2014, Springer, 327–352.

[101] Ingerslev H. C. , Fish Health and Fish Quality: Effects of Tissue Regeneration From a Molecular Perspective, 2010, Technical University of Denmark.

[102] Schmidt J. G. , Nielsen M. E. , and Ersbøll B. K. , Wound Healing in Rainbow Trout (*Oncorhynchus mykiss*) and Common Carp (*Cyprinus carpio*), 2013. 10.1016/j.fsi.2015.12.010 26702558

[103] Klasing K. C. , Adler K. L. , Calvert C. C. , and Remus J. C. , Dietary Betaine Increases Intraepithelial Lymphocytes in the Duodenum of Coccidia-Infected Chicks and Increases Functional Properties of Phagocytes, The Journal of Nutrition. (2002) 132, no. 8, 2274–2282, 10.1093/jn/132.8.2274. 12163675

[104] Muona M. and Virtanen E. , Effect of Dimethylglycine and Trimethylglycine (Betaine) on the Response of Atlantic Salmon (*Salmo salar* L.) Smolts to Experimental Vibrio Anguillarum Infection, Fish & Shellfish Immunology. (1993) 3, no. 6, 439–449, 10.1006/fsim.1993.1043, 2-s2.0-0027145935.

[105] Aderem A. and Underhill D. M. , Mechanisms of Phagocytosis in Macrophages, Annual Review of Immunology. (1999) 17, no. 1, 593–623, 10.1146/annurev.immunol.17.1.593, 2-s2.0-0033046220. 10358769

[106] Havixbeck J. and Barreda D. , Neutrophil Development, Migration, and Function in Teleost Fish, Biology. (2015) 4, no. 4, 715–734, 10.3390/biology4040715, 2-s2.0-84946820115. 26561837 PMC4690015

[107] Warskulat U. , Zhang F. , and Häussinger D. , Modulation of Phagocytosis by Anisoosmolarity and Betaine in Rat Liver Macrophages (Kupffer Cells) and RAW 264.7 Mouse Macrophages, FEBS Letters. (1996) 391, no. 3, 287–292, 10.1016/0014-5793(96)00753-3, 2-s2.0-0030581297. 8764991

[108] El-Benna J. , Hurtado-Nedelec M. , Marzaioli V. , Marie J.-C. , Gougerot-Pocidalo M.-A. , and Dang P. M.-C. , Priming of the Neutrophil Respiratory Burst: Role in Host Defense and Inflammation, Immunological Reviews. (2016) 273, no. 1, 180–193, 10.1111/imr.12447, 2-s2.0-84983490033. 27558335

[109] Fujiwara N. and Kobayashi K. , Macrophages in Inflammation, Current Drug Target –Inflammation & Allergy. (2005) 4, no. 3, 281–286, 10.2174/1568010054022024, 2-s2.0-21044449439. 16101534

[110] Kilercioğlu S. , Fish Immune System, Mucosal Immunity and Functions of IL-1*β*, TNF-*α* and IL-18 Proinflammatory Cytokines, 2021.

[111] Kim D. H. , Sung B. , and Kang Y. J. , et al.Anti-Inflammatory Effects of Betaine on AOM/DSS Induced Colon Tumorigenesis in ICR Male Mice, International Journal of Oncology. (2014) 45, no. 3, 1250–1256, 10.3892/ijo.2014.2515, 2-s2.0-84904394027. 24969167

[112] Xu J. , Nie Z. , Qiu X. , Zhang J. , and Han S. , Effects of Betaine Supplementation on Inflammatory Markers: A Systematic Review and Meta-Analysis of Randomised Controlled Trials, International Journal of Food Sciences and Nutrition. (2023) 74, no. 7, 721–729, 10.1080/09637486.2023.2257906. 37733077

[113] Platzer C. , Interleukin-10: An Anti-Inflammatory and Immunosuppressive Cytokine in the Normal and Pathological Immune Response, Current Medicinal Chemistry - Anti-Inflammatory & Anti-Allergy Agents. (2003) 2, no. 4, 309–323, 10.2174/1568014033483653.

[114] Peake J. and Suzuki K. , Neutrophil Activation, Antioxidant Supplements and Exercise-Induced Oxidative Stress, Exercise Immunology Review. (2004) 10, no. 1, 129–141. 15633591

[115] Kirkham P. , Oxidative Stress and Macrophage Function: A Failure to Resolve the Inflammatory Response, 2007. 10.1042/BST0350284 17371261

[116] Johnson J. , Jaggers R. M. , and Gopalkrishna S. , et al.Oxidative Stress in Neutrophils: Implications for Diabetic Cardiovascular Complications, Antioxidants & Redox Signaling. (2022) 36, no. 10–12, 652–666, 10.1089/ars.2021.0116. 34148367 PMC9057880

[117] Zhang M. , Zhang H. , and Li H. , et al.Antioxidant Mechanism of Betaine Without Free Radical Scavenging Ability, Journal of Agricultural and Food Chemistry. (2016) 64, no. 42, 7921–7930, 10.1021/acs.jafc.6b03592, 2-s2.0-84992740858. 27677203

[118] Denkert C. , Warskulat U. , Hensel F. , and Häussinger D. , Osmolyte Strategy in Human Monocytes and Macrophages: Involvement of p38MAPKin Hyperosmotic Induction of Betaine and Myoinositol Transporters, Archives of Biochemistry and Biophysics. (1998) 354, no. 1, 172–180, 10.1006/abbi.1998.0661, 2-s2.0-0031858356. 9633613

[119] Kato T. and Kitagawa S. , Regulation of Neutrophil Functions by Proinflammatory Cytokines, International Journal of Hematology. (2006) 84, no. 3, 205–209, 10.1532/IJH97.06141, 2-s2.0-33750327298. 17050192

[120] Go E. K. , Jung K. J. , and Kim J. M. , et al.Betaine Modulates Age-Related NF-*κ*B by Thiol-Enhancing Action, Biological and Pharmaceutical Bulletin. (2007) 30, no. 12, 2244–2249, 10.1248/bpb.30.2244, 2-s2.0-36849014318. 18057706

[121] Liu T. , Zhang L. , Joo D. , and Sun S.-C. , NF-*κ*B Signaling in Inflammation, Signal Transduction and Targeted Therapy. (2017) 2, no. 1, 1–9, 10.1038/sigtrans.2017.23, 2-s2.0-85064627519. PMC5661633 29158945

[122] Go E. K. , Jung K. J. , Kim J. Y. , Yu B. P. , and Chung H. Y. , Betaine Suppresses Proinflammatory Signaling During Aging: The Involvement of Nuclear Factor-*κ*B via Nuclear Factor-Inducing Kinase/I*κ*B Kinase and Mitogen-Activated Protein Kinases, The Journals of Gerontology Series A: Biological Sciences and Medical Sciences. (2005) 60, no. 10, 1252–1264, 10.1093/gerona/60.10.1252, 2-s2.0-27744467465. 16282556

[123] Manzoor Z. and Koh Y.-S. , Mitogen-Activated Protein Kinases in Inflammation, Journal of Bacteriology and Virology. (2012) 42, no. 3, 189–195, 10.4167/jbv.2012.42.3.189, 2-s2.0-84922685657.

[124] Huang G. , Shi L. Z. , and Chi H. , Regulation of JNK and p38 MAPK in the Immune System: Signal Integration, Propagation and Termination, Cytokine. (2009) 48, no. 3, 161–169, 10.1016/j.cyto.2009.08.002, 2-s2.0-70350028721. 19740675 PMC2782697

[125] Weichhart T. and Säemann M. D. , The PI3K/Akt/mTOR Pathway in Innate Immune Cells: Emerging Therapeutic Applications, Annals of the Rheumatic Diseases. (2008) 67, no. Suppl 3, iii70–iii74, 10.1136/ard.2008.098459, 2-s2.0-56749156696. 19022819

[126] Xie S. , Chen M. , and Yan B. , et al.Identification of a Role for the PI3K/AKT/mTOR Signaling Pathway in Innate Immune Cells, PLoS ONE. (2014) 9, no. 4, 10.1371/journal.pone.0094496, 2-s2.0-84899546121, e94496. 24718556 PMC3981814

[127] Pompura S. L. and Dominguez-Villar M. , The PI3K/AKT Signaling Pathway in Regulatory T-Cell Development, Stability, and Function, Journal of Leukocyte Biology. (2018) 103, no. 6, 1065–1076, 10.1002/JLB.2MIR0817-349R, 2-s2.0-85050089962. 29357116

[128] Zhou T. , Shi J. , and Li X. , Role of PI3K/Akt Signaling Pathway in the Innate Immune of Sepsis, Zhonghua Wei Zhong Bing Ji Jiu Yi Xue. (2018) 30, no. 11, 1091–1094. 30541652 10.3760/cma.j.issn.2095-4352.2018.011.016

[129] Veskovic M. , Mladenovic D. , and Milenkovic M. , et al.Betaine Modulates Oxidative Stress, Inflammation, Apoptosis, Autophagy, and Akt/mTOR Signaling in Methionine-Choline Deficiency-Induced Fatty Liver Disease, European Journal of Pharmacology. (2019) 848, 39–48, 10.1016/j.ejphar.2019.01.043, 2-s2.0-85060871124. 30689995

[130] Zhang H. , Nie L. , and Qin K. , et al.Betaine Induces Apoptosis of C4-2B Prostate Cancer Cells via Inhibiting PI3K/AKT/NF-*κ*B Signaling Pathway, Xi Bao Yu Fen Zi Mian Yi Xue Za Zhi = Chinese Journal of Cellular and Molecular Immunology. (2021) 37, no. 6, 513–519. 34060446

[131] Waickman A. T. and Powell J. D. , mTOR, Metabolism, and the Regulation of T-Cell Differentiation and Function, Immunological Reviews. (2012) 249, no. 1, 43–58, 10.1111/j.1600-065X.2012.01152.x, 2-s2.0-84865301337. 22889214 PMC3419491

[132] Linke M. , Fritsch S. D. , Sukhbaatar N. , Hengstschläger M. , and Weichhart T. , mTORC 1 and mTORC 2 as Regulators of Cell Metabolism in Immunity, FEBS Letters. (2017) 591, no. 19, 3089–3103, 10.1002/1873-3468.12711, 2-s2.0-85021293508. 28600802 PMC6322652

[133] Saxton R. A. and Sabatini D. M. , mTOR Signaling in Growth, Metabolism, and Disease, Cell. (2017) 168, no. 6, 960–976, 10.1016/j.cell.2017.02.004, 2-s2.0-85014844261. 28283069 PMC5394987

[134] Howell J. J. , Ricoult S. J. , Ben-Sahra I. , and Manning B. D. , A Growing Role for mTOR in Promoting Anabolic Metabolism, Biochemical Society Transactions. (2013) 41, no. 4, 906–912, 10.1042/BST20130041, 2-s2.0-84880566446. 23863154

[135] Polak P. and Hall M. N. , mTOR and the Control of Whole Body Metabolism, Current Opinion in Cell Biology. (2009) 21, no. 2, 209–218, 10.1016/j.ceb.2009.01.024, 2-s2.0-63749105226. 19261457

[136] Pfitzner E. , Kliem S. , Baus D. , and Litterst M. C. , The Role of STATs in Inflammation and Inflammatory Diseases, Current Pharmaceutical Design. (2004) 10, no. 23, 2839–2850, 10.2174/1381612043383638, 2-s2.0-4444281108. 15379672

[137] Egwuagu C. E. , STAT3 in CD4+ T Helper Cell Differentiation and Inflammatory Diseases, Cytokine. (2009) 47, no. 3, 149–156, 10.1016/j.cyto.2009.07.003, 2-s2.0-68949131070. 19648026 PMC2733795

[138] Jin Y. , Zhou T. , and Li N. , et al.JAK and STAT Members in Channel Catfish: Identification, Phylogenetic Analysis and Expression Profiling After *Edwardsiella ictaluri* Infection, Developmental & Comparative Immunology. (2018) 81, 334–341, 10.1016/j.dci.2017.12.019, 2-s2.0-85039761659. 29274790

[139] Rao S. S. , Nelson P. A. , Lunde H. S. , and Haugland G. T. , Evolutionary, Comparative, and Functional Analyses of STATs and Regulation of the JAK-STAT Pathway in Lumpfish Upon Bacterial and Poly (I:C) Exposure, Frontiers in Cellular and Infection Microbiology. (2023) 13, 10.3389/fcimb.2023.1252744, 1252744. 37808912 PMC10556531

[140] Ji Y. , Gao S. , Feng X. , and He L. , Calcium Channel Mechanism by Which Betaine Promotes Proliferation of Lymphocytes in Mice, Zhongguo Zhong Yao Za Zhi = Zhongguo Zhongyao Zazhi = China Journal of Chinese Materia Medica. (2009) 34, no. 15, 1959–1963. 19894544

[141] Díaz-Rosales P. , Muñoz-Atienza E. , and Tafalla C. , Role of Teleost B Cells in Viral Immunity, Fish & Shellfish Immunology. (2019) 86, 135–142, 10.1016/j.fsi.2018.11.039, 2-s2.0-85056749807. 30448446

[142] Xiuan Z. , Effects of Betaine on Immune Responses of Newcastle Disease in Broiler Chicks, Zhongguo Shou Yi Xue Bao = Chinese Journal of Veterinary Science. (2001) 21, no. 1, 99–102.

[143] Huang B. , Hu X. , Hu J. , Chen Z. , and Zhao H. , Betaine Alleviates Cognitive Deficits in Diabetic Rats via PI3K/Akt Signaling Pathway Regulation, Dementia and Geriatric Cognitive Disorders. (2020) 49, no. 3, 270–278, 10.1159/000508624. 32702702

[144] Larbi A. , Kempf J. , and Pawelec G. , Oxidative Stress Modulation and T Cell Activation, Experimental Gerontology. (2007) 42, no. 9, 852–858, 10.1016/j.exger.2007.05.004, 2-s2.0-34547954316. 17604927

[145] Alirezaei M. , Khoshdel Z. , Dezfoulian O. , Rashidipour M. , and Taghadosi V. , Beneficial Antioxidant Properties of Betaine against Oxidative Stress Mediated by Levodopa/Benserazide in the Brain of Rats, The Journal of Physiological Sciences. (2015) 65, no. 3, 243–252, 10.1007/s12576-015-0360-0, 2-s2.0-84939993002. 25665954 PMC10717468

[146] Biller J. D. and Takahashi L. S. , Oxidative Stress and Fish Immune System: Phagocytosis and Leukocyte Respiratory Burst Activity, Anais da Academia Brasileira de Ciências. (2018) 90, no. 4, 3403–3414, 10.1590/0001-3765201820170730, 2-s2.0-85059812418. 30365708

[147] Chen Y. , Zhou Z. , and Min W. , Mitochondria, Oxidative Stress and Innate Immunity, Frontiers in Physiology. (2018) 9, 10.3389/fphys.2018.01487, 2-s2.0-85055286673, 1487. 30405440 PMC6200916

[148] Chowdhury S. and Saikia S. K. , Oxidative Stress in Fish: A Review, Journal of Scientific Research. (2020) 12, no. 1, 145–160, 10.3329/jsr.v12i1.41716.

[149] Hoseinifar S. H. , Yousefi S. , and Van Doan H. , et al.Oxidative Stress and Antioxidant Defense in Fish: The Implications of Probiotic, Prebiotic, and Synbiotics, Reviews in Fisheries Science & Aquaculture. (2021) 29, no. 2, 198–217, 10.1080/23308249.2020.1795616.

[150] Sun S. , Li B. , and Wu M. , et al.Effect of Dietary Supplemental Vitamin C and Betaine on the Growth Performance, Humoral Immunity, Immune Organ Index, and Antioxidant Status of Broilers Under Heat Stress, Tropical Animal Health and Production. (2023) 55, no. 2, 10.1007/s11250-023-03500-y, 96. 36823253

[151] Yu L. , Jin Y. , Cui H. , Luo Y. , Dong L. , and Wang H. , Effects of Dietary Rumen-Protected Betaine Supplementation on the Antioxidant Status of Lambs, Livestock Science. (2020) 237, 10.1016/j.livsci.2020.104026, 104026.

[152] El-Shater S. N. , Abo-EL-Sooud K. , and Tolba A. , et al.Effect of in-Ovo Inoculation of Betaine on Hatchability, Serum Antioxidant Levels, Muscle Gene Expression and Intestinal Development of Broiler Chicks, Journal of Animal Physiology and Animal Nutrition. (2024) 108, no. 4, 883–890, 10.1111/jpn.13938. 38353323

[153] Chen R. , Yang M. , and Song Y. D. , et al.Effect of Anhydrous Betaine and Hydrochloride Betaine on Growth Performance, Meat Quality, Postmortem Glycolysis, and Antioxidant Capacity of Broilers, Poultry Science. (2022) 101, no. 4, 10.1016/j.psj.2021.101687, 101687. PMC8844660 35139439

[154] Zhang M. , Wang T. , Ou S. , Zou Y. , and Xin X. , Betaine Activates the Nrf2-Keap1-ARE Pathway by Increasing the Methylation Level of Keap1 DNA Promoter, International Journal of Food Science and Technology. (2024) 59, no. 9, 6231–6242, 10.1111/ijfs.17359.

[155] Coban J. , Bingul I. , Yesil-Mizrak K. , Dogru-Abbasoglu S. , Oztezcan S. , and Uysal M. , Effects of Carnosine Plus Vitamin E and Betaine Treatments on Oxidative Stress in Some Tissues of Aged Rats, Current Aging Science. (2013) 6, no. 2, 199–205, 10.2174/18746098112059990011, 2-s2.0-84882781778. 23701646

[156] Attia Y. A. , El-Naggar A. S. , Abou-Shehema B. M. , and Abdella A. A. , Effect of Supplementation With Trimethylglycine (Betaine) and/or Vitamins on Semen Quality, Fertility, Antioxidant Status, DNA Repair and Welfare of Roosters Exposed to Chronic Heat Stress, Animals. (2019) 9, no. 8, 10.3390/ani9080547, 2-s2.0-85071265400, 547. 31408981 PMC6719041

[157] Hassanen E. I. , Hassan N. H. , Hussien A. M. , Ibrahim M. A. , and Ali M. E. , Betaine Alleviates Methomyl-Triggered Oxidative Stress-Mediated Cardiopulmonary Inflammation in Rats Through iNOS/Cox2 and Nrf2/HO1/Keap1 Signaling Pathway, Toxicology and Applied Pharmacology. (2025) 495, 10.1016/j.taap.2024.117223, 117223. 39742927

[158] Ratriyanto A. , Mosenthin R. , Bauer E. , and Eklund M. , Metabolic, Osmoregulatory and Nutritional Functions of Betaine in Monogastric Animals, Asian-Australasian Journal of Animal Sciences. (2009) 22, no. 10, 1461–1476, 10.5713/ajas.2009.80659, 2-s2.0-72549094358.

[159] Wargo M. J. , Homeostasis and Catabolism of Choline and Glycine Betaine: Lessons From *Pseudomonas aeruginosa* , Applied and Environmental Microbiology. (2013) 79, no. 7, 2112–2120, 10.1128/AEM.03565-12, 2-s2.0-84875520938. 23354714 PMC3623244

[160] Gomez-Jimenez S. , Valenzuela-Soto E. M. , Zamorano-Apodaca J. C. , Gamez-Alejo L. A. , and Muñoz-Bacasehua C. , Glycine Betaine Levels and BADH Activity of Juvenile Shrimp *Litopenaeus vannamei* in Response to Vibrio Bacterial Infection and Sudden Hyperosmotic Stress, Aquaculture Journal. (2025) 5, no. 1, 10.3390/aquacj5010004, 4.

[161] He G.-L. , Shi M.-L. , and Liu Y.-C. , et al.Effects of Dietary Betaine Supplementation on Growth Performance, Feed Intake, Intestinal Histology, Lipid Metabolism, and Immune Response of Black Tiger Shrimp (*Penaeus monodon*) Fed Diets Containing Two Levels of Raw Feed Attractants, Aquaculture International. (2024) 32, no. 1, 653–673, 10.1007/s10499-023-01180-x.

[162] Robinson M. W. , Harmon C. , and O’Farrelly C. , Liver Immunology and Its Role in Inflammation and Homeostasis, Cellular & Molecular Immunology. (2016) 13, no. 3, 267–276, 10.1038/cmi.2016.3, 2-s2.0-84966311104. 27063467 PMC4856809

[163] Uribe C. , Folch H. , Enríquez R. , and Moran G. J. V. M. , Innate and Adaptive Immunity in Teleost Fish: A Review, Veterinární Medicína. (2011) 56, no. 10, 486–503, 10.17221/3294-VETMED, 2-s2.0-84855725332.

[164] Ahmad I. , Irm M. , and Ahmed I. , et al.Role of Ginger in Fish Nutrition With Special Emphasis on Growth, Health, Gut and Liver Morphology, Journal of the World Aquaculture Society. (2024) 55, no. 6, 10.1111/jwas.13101, e13101.

[165] Jin Y. , Zheng S. , and Pu Y. , et al.Cypermethrin Has the Potential to Induce Hepatic Oxidative Stress, DNA Damage and Apoptosis in Adult Zebrafish (*Danio rerio*), Chemosphere. (2011) 82, no. 3, 398–404, 10.1016/j.chemosphere.2010.09.072, 2-s2.0-78650072640. 20965546

[166] Stoliar O. B. and Lushchak V. I. , Environmental Pollution and Oxidative Stress in Fish, Oxidative Stress-Environmental Induction and Dietary Antioxidants, 2012, IntechOpen, 131–166.

[167] Hajam M. E. , Plavan G.-I. , and Kandri N. I. , et al.Evaluation of Softwood and Hardwood Sawmill Wastes Impact on the Common Carp (*Cyprinus carpio*) and Its Aquatic Environment: An Oxidative Stress Study, Environmental Toxicology and Pharmacology. (2020) 75, 10.1016/j.etap.2020.103327, 103327. 31924571

[168] Topić Popović N. , Čižmek L. , Babić S. , Strunjak-Perović I. , and Čož-Rakovac R. , Fish Liver Damage Related to the Wastewater Treatment Plant Effluents, Environmental Science and Pollution Research. (2023) 30, no. 17, 48739–48768, 10.1007/s11356-023-26187-y. 36869954 PMC9985104

[169] Shiry N. , Alavinia S. J. , Impellitteri F. , Alavinia S. J. , and Faggio C. , Beyond the Surface: Consequences of Methyl Tert-Butyl ether (MTBE) Exposure on Oxidative Stress, Haematology, Genotoxicity, and Histopathology in Rainbow Trout, Science of the Total Environment. (2023) 900, 10.1016/j.scitotenv.2023.165784, 165784. 37499819

[170] Banaee M. , Di Paola D. , Cuzzocrea S. , Cordaro M. , and Faggio C. , Biochemical and Physiological Response During Oxidative Stress—From Invertebrates to Vertebrates, 2024, IntechOpen.

[171] Heidari R. , Niknahad H. , and Sadeghi A. , et al.Betaine Treatment Protects Liver Through Regulating Mitochondrial Function and Counteracting Oxidative Stress in Acute and Chronic Animal Models of Hepatic Injury, Biomedicine & Pharmacotherapy. (2018) 103, 75–86, 10.1016/j.biopha.2018.04.010, 2-s2.0-85045040706. 29635131

[172] Barak A. J. , Beckenhauer H. C. , and Tuma D. J. , Betaine, Ethanol, and the Liver: A Review, Alcohol. (1996) 13, no. 4, 395–398, 10.1016/0741-8329(96)00030-4, 2-s2.0-0030199376. 8836329

[173] Cheng H. L. , Xia D. Q. , and Wu T. T. , Fatty Liver and Regulation of Lipids Metabolism in Fish, Chinese Journal of Animal Nutrition. (2006) 18, 294–298.

[174] Forn-Cuní G. , Varela M. , Fernández-Rodríguez C. M. , Figueras Huerta A. , and Novoa B. , Liver Immune Responses to Inflammatory Stimuli in a Dietinduced Obesity Model of Zebrafish, Journal of Endocrinology. (2015) 224, no. 2, 159–170. 25371540 10.1530/JOE-14-0398

[175] Ruiz-Ramírez J. A. , Ramírez-Ayala E. , and Tintos-Gómez A. , et al.Hepatocellular Steatosis as a Response to Nutritional Stressors in *Lutjanus guttatus* (Steindachner, 1869) Grown in Floating Cages: A Case Study, Latin American Journal of Aquatic Research. (2019) 47, no. 4, 709–715, 10.3856/vol47-issue4-fulltext-14, 2-s2.0-85073777471.

[176] Obeid R. , The Metabolic Burden of Methyl Donor Deficiency With Focus on the Betaine Homocysteine Methyltransferase Pathway, Nutrients. (2013) 5, no. 9, 3481–3495, 10.3390/nu5093481, 2-s2.0-84884175034. 24022817 PMC3798916

[177] Möller A.-M. , Korytář T. , Köllner B. , Schmidt-Posthaus H. , and Segner H. , The Teleostean Liver as an Immunological Organ: Intrahepatic Immune Cells (IHICs) in Healthy and Benzo[a]pyrene Challenged Rainbow Trout (*Oncorhynchus mykiss*), Developmental & Comparative Immunology. (2014) 46, no. 2, 518–529, 10.1016/j.dci.2014.03.020, 2-s2.0-84904430465. 24718255

[178] Ardeshir R. A. , Movahedinia A. A. , and Rastgar S. , Fish Liver Biomarkers for Heavy Metal Pollution: A Review Article, American Journal of Toxicology. (2017) 2, no. 1, 1–8.

[179] Causey D. R. , Pohl M. A. N. , Stead D. A. , Martin S. A. M. , Secombes C. J. , and Macqueen D. J. , High-Throughput Proteomic Profiling of the Fish Liver Following Bacterial Infection, BMC Genomics. (2018) 19, no. 1, 1–17, 10.1186/s12864-018-5092-0, 2-s2.0-85054242839. 30285610 PMC6167799

[180] González-Silvera D. , Cuesta A. , and Esteban M.Á. , Immune Defence Mechanisms Presented in Liver Homogenates and Bile of Gilthead Seabream (*Sparus aurata*), Journal of Fish Biology. (2021) 99, no. 6, 1958–1967, 10.1111/jfb.14901. 34486119

[181] Mahmoud A. M. and Ali M. M. , Methyl Donor Micronutrients That Modify DNA Methylation and Cancer Outcome, Nutrients. (2019) 11, no. 3, 10.3390/nu11030608, 2-s2.0-85062974863, 608. 30871166 PMC6471069

